# Neuronal injury and death following focal mild brain injury: The role of network excitability and seizure

**DOI:** 10.22038/IJBMS.2019.37558.8932

**Published:** 2020-01

**Authors:** Tahereh Ghadiri, Ali Gorji, Gelareh Vakilzadeh, Vahid Hajali, Fariba Khodagholi, Mohammad Sharifzadeh

**Affiliations:** 1 Department of Neurosciences, Faculty of Advanced Medical Sciences, Tabriz University of Medical Sciences, Tabriz, Iran; 2 Shefa Neuroscience Research Center, Khatamolanbia Hospital, Tehran , Iran; 3 Department of Neurosurgery, Department of Neurology, and Epilepsy Research Center, Munster University, Germany; 4 Razavi Neuroscience Center, Razavi Hospital, Mashhad, Iran; 5 Quchan Higher Health Education Center, Mashhad University of Medical Sciences, Mashhad, Iran; 6 Neuroscience Research Center , Shahid Beheshti University of Medical Sciences, Tehran, Iran; 7 Faculty of Pharmacy, Department of Toxicology, Tehran University of Medical Sciences, Tehran, Iran

**Keywords:** Apoptosis, Convulsive, Hippocampus, Neuroinflammation, Post-traumatic, Seizure

## Abstract

**Objective(s):**

While traumatic brain injury (TBI) is a predisposing factor for development of post-traumatic epilepsy (PTE), the occurrence of seizures following brain trauma can infuriate adverse consequences of brain injury. However, the effect of seizures in epileptogenesis after mild TBI cannot yet be accurately confirmed. This study was designed to investigate the histopathological and molecular modifications induced by seizures on traumatized brain.

**Materials and Methods:**

Using a new method, head was traumatized and seizures were evoked by sub-convulsive dose of pentylenetetrazole (PTZ) fifteen days after induction of focal mild TBI. Convulsion assessments were performed one hour after PTZ injection and was followed by histopathological and molecular evaluations.

**Results:**

A significantly higher score and longer duration of seizure attacks as well as higher number of epileptiform discharges were observed in the TBI+PTZ group compared to sham and TBI groups. An elevated number of apoptotic cells was observed in the TBI+PTZ group compared to sham and TBI rats. Molecular investigations revealed higher levels of Bax/Bcl2 ratio, Caspase 3, and NF-κB in the TBI+PTZ group compared to the other animal groups. The value of Nrf2 did not change after mild TBI compared to sham and PTZ control groups. Occurrence of seizures after TBI, however, significantly decreased the level of Nrf2.

**Conclusion:**

Our data indicated that seizure occurrence following mild TBI aggravates cell injury and death via activation of neuroinflammatory processes and may increase the risk of PTE. Additionally, our results suggest a potential protective role of Nrf2 after chemically evoked PTE.

## Introduction

Traumatic brain injury (TBI) can lead to life-long complications, such as epilepsy (1). The risk of developing epilepsy after TBI ranges from 1.6% to 4.2% after moderate TBI and from 25.3% to 53% after severe TBI with local brain lesions (2, 3) or penetrating head wounds (4-6). It is clear that the occurrence of seizures after TBI is also correlated to the severity of brain trauma. 

Pathophysiology of TBI is complicated and includes immediate primary damage of brain tissue due to direct mechanical insult and secondary injury processes that lead to various forms of neuronal cell death and synaptic loss (7). A silent interval (the presumed period of epileptogenesis) between the head trauma and early acute seizures and the appearance of spontaneous recurrent seizures after TBI has been reported in different animal models of epilepsy (8, 9). Numerous histopathological changes such as neuronal and synaptic hyperactivity, blood-brain barrier damage, apoptosis, neurogenesis, gliosis, axonal damage, activation of inflammatory processes, alterations of the extracellular matrix, axonal sprouting, and reorganization of the neuronal network were reported during this silent period (8-10). However, these alterations are not unique for TBI and have also been observed after epileptic seizures (11). 

Hyperexcitability of the neuronal network after moderate to severe TBI has been shown in the cerebral cortex and hippocampus (12). One explanation for such increased seizure susceptibity following truama is the divergent vulnerability of neurons to brain insults that results in selective death among neuronal populations during the secondary injury of TBI (13-16). Therefore, this phase of damage may affect specific neuronal circuits and perturb balance between inhibitory and excitatory connections in favor of evoking spontaneous seizures (17). Accordingly, an animal study revealed that a non-convulsant dose of pentylenetetrazole (PTZ), a GABAA receptor antagonist, induced generalized tonic-clonic seizures 15 weeks after injury (18). 

In addition, post-injury administration of a single low dose of kainic acid, a Glutamate agonist, augmented hippocampal glucose metabolic rates and resulted in extensive neuronal death in the ipsilateral hippocampal CA3, CA4, and hilar regions (19). In another study, an induced period of post-traumatic epilepsy (PTE) using PTZ remarkably worsened the cellular damage following TBI (7). These observations suggest that seizure activity after TBI may exacerbate the outcome and exaggerate the primary insult (7, 12). In the clinic, the occurrence of PTE is also associated with severe TBI, cognitive deficit, and hippocampal atrophy (12, 20). Development of seizure attacks early after TBI enhanced seizure susceptibility and decreased seizure threshold in several models of TBI (18, 19, 21-24). This is worth mentioning that, the structural consequences of TBI including cellular and synaptic reorganization occur during the period of time (days to months), and thus the potential role of the double insult of a PTE on neuronal vulnerability and its consequences can be investigated (12). 

In the present study, we investigated molecular and cellular outcomes following induced PTE. In order to produce disease model, animals received a mild focal brain injury (25) or sham surgery, and 2 weeks later, seizure behavior was assessed by challenging the animals with PTZ and scored using a clinical grading scale (7). This research aimed to identify alterations in apoptotic and inflammatory mediators after induction of PTE in the rat brain.

## Materials and Methods

***Animals***

Forty-eight male Wistar rats were housed in a 12 hr light and dark cycle with free access to food and water. All experiments were carried out according to the laboratory animal research guidelines of the ethics committee of Shefa Neuroscience Research Center, Tehran, Iran.

The rats were randomly assigned into 4 groups: (i) Sham: animals were only subjected to skin incision without any other intervention (n = 12); (ii) PTZ: animals were intraperitoneally injected with a sub-convulsive dose of PTZ (30 mg/kg, n = 12); (iii) TBI: animals received intraperitoneal injections of saline 15 days after mild TBI (n = 12); (IV) TBI+PTZ: animals received PTZ (30 mg/kg) 15 days after a mild TBI (n = 12). All sham and PTZ animals were age-matched with TBI and TBI+PTZ rats for all experiments.

***Electrode implantation and induction of mild TBI***

A detailed description of our method and an assessment of its accuracy have been given previously (25). Briefly, rats were anesthetized by intraperitoneal injection of a mixture of ketamine (100 mg/ml, Sigma, USA) and xylazine (20 mg/ml, Rompun, Bayer, Germany) throughout all surgical procedures and TBI (26). The skull was shaved and antiseptically cleaned before positioning the animal in a stereotaxic frame. Then, skull periosteum was exposed by a midline longitudinal scalp incision after securing in a stereotaxics and the fascia cleared to expose the surface of the skull. Two silver cortical electrodes were inserted over the left and the right somatosensory cortices (2 mm lateral and 3 mm rostral to bregma). Two additional electrodes were placed into the skull over the nasal bone (rostral to bregma, 5 mm lateral to the midline), served as a reference electrode. A focal brain injury in the right parietal cortex was caused by a given weight under stereotaxic guidance. The device could hold up to 500 g and the force was exerted through a thin metal tip onto the skull of the animal. While the head was fixed in a stereotaxic frame, the short arm of the device, carrying 500 g of weight, was released from 30 cm distance at a 65° angle on the right parietal cortex. The surface of the tip of the hammer-like metal rod was 4 mm2 and the impact force of each weight drop was 17.2 N. After TBI, the rats received supporting oxygenation during the recovery period. Following TBI, the entire area was cemented by dental acrylic.

***Neurological deficit severity evaluation***

The severity of neurological impairments was assessed at various time intervals after TBI (1, 24, and 48 hr, as well as 7 and 14 days), using a modified Neurological Severity Score (mNSS) (27).

***PTZ challenging test***

To detect seizure susceptibility following TBI, animals underwent PTZ treatment by intraperitoneal injection of a sub-convulsive dose of PTZ (30 mg/kg in 0.9% saline; Sigma, St.Louis, MO) 2 weeks after TBI. Following the PTZ injection, each rat was placed in a transparent plastic cage and observed for 60 min. The latency for behavioral seizure activity was defined as the time lag between PTZ injection and the occurrence of the first tonic-clonic activity. Convulsions were scored into five classes by a standard method as follows: Class 1, myoclonic (brief jerks of a muscle or a group of muscles); Class 2, unilateral clonic (<1 min); Class 3, bilateral clonic (<1 min); Class 4, bilateral clonic sustained (>1min); Class 5, tonic-clonic (severe muscles contractions with loss of postural control) (28).

***Electrocorticographical assessment***

 To detect any hyperexitibility, electrical activity of the brain was recorded under sedation by chloral hydrate (400 mg/kg, Sigma, St. Louis, USA) (29). Recording electrodes were stereotaxically implanted on the dura mater of the left and right somatosensory cortices. Electrocorticogram (ECoG) was recorded by silver electrodes connected to an amplifier (EXT-02 F, NPI, Germany) and sent to a computer (IBM, USA) through the digital-analog converter (Digidata 1440A digitizer; Axon CNS Instrument). ECoG recordings were performed for 10 min before and one hour after PTZ (30 mg/kg,IP; Sigma, dissolved in saline) injection. Latency, frequency, amplitude, and duration of epileptiform activities were extracted and calculated using AxoScope 10.2 software (Axon; Molecular Devices). Spike-and-wave complex (< 15 s) was considered as epileptiform activities (30), and the number of spike and wave discharges (SWDs; epileptiform discharge) were counted during 60 min after injection of a single sub-convulsant dose of PTZ.

***Histopathology and DNA fragmentation analysis***

Following the induction of seizures by PTZ, animals were sacrificed and, after transcardial perfusion, brains were removed and embedded in paraffin. In situ labeling of DNA fragmentation using a terminal deoxynucleotidyl-transferase-mediated dUTP nick-end labeling (TUNEL) Kit (Roche, Mannheim, Germany) was performed on brain sections, as previously described (31). Briefly, three coronal 8 μm thick sections (between -3.2 to -4.00 mm from the bregma) from each block were dewaxed and dehydrated by heating to 60 °C, followed by washing in xylene and rehydration through washing with diluted alcohol. After being washed with 10 mM Tris-HCl (pH 7.6), the sections were incubated in methanol containing 0.3 % H_2_O_2_ for 10 min to inhibit endogenous peroxidase activity. They were then treated with proteinase K (Roche, 20 μg/ml in Tris buffer) at 37 °C for 30 min. The sections were incubated in TUNEL reaction mixture at 37 °C for 60 min and in POD solution for 30 min. The color reaction was developed in 3-3′-diaminobenzidine (DAB, Roche; 0.5 μl DAB and 1.5 μl peroxide buffer) for 5–10 min and counterstaining was performed with Cresyl violet. The extent of brain damage was evaluated as an average number of TUNEL-positive cells in each section viewed under 40 × magnification and counted in 10 microscopic fields from the parietal cortex, hippocampal CA1, and CA3 ipsilateral to the TBI site.

***Western blotting***

The level of proteins was evaluated by the western blot method. At the end of the seizures, brains were removed after transcardial perfusion of 150 ml cold phosphate-buffered saline. The right hippocampus (of the traumatized hemisphere, n=4) was extracted. Hippocampal specimens after homogenization were lysed in buffer containing a protease phosphatase inhibitor cocktail. Protein concentrations were determined according to Bradford’s method (32). The total proteins were electrophoresed on 12% SDS-PAGE gels, transferred to polyvinylidene fluoride (PVDF) membranes, and detected by nuclear factor (erythroid-derived 2)-like 2 (Nrf2) (Abcam, Cambridge, MA, USA), Caspase 3 (Santa Cruz, Germany), necrosis factor kappa B (NF-κB), Bcl2-associated x protein (Bax), and B cell lymphoma 2 (Bcl2) (Cell Signaling, Beverly, MA, USA) antibodies. Immunoreactive polypeptides were illuminated using enhanced chemiluminescence (ECL) reagents (Amersham Bioscience, USA) and subsequent radiography. Data analysis was performed using ImageJ software (Version 1.46, National Institutes of Health) by measuring the integrated density of bands after background subtraction on films (33).

***Statistical analysis***

Statistical analyses were performed with (GraphPad Software, Inc., San Diego, CA). Latency and duration of PTZ-induced seizures were expressed as the average time (min) for the first generalized seizure corresponding to scores V, and for the occurrence of seizures were compared by means of a one-way analysis of variance (ANOVA) test followed by Newman–Keuls test as appropriate. Latency in the ECoG recordings was expressed as the average time to the first SWD. All values were expressed as mean±SEM (standard error of the mean). A *P* value of 0.05 or less was considered significant.

## Results

***Neurologic impairments***

**Figure 1:**
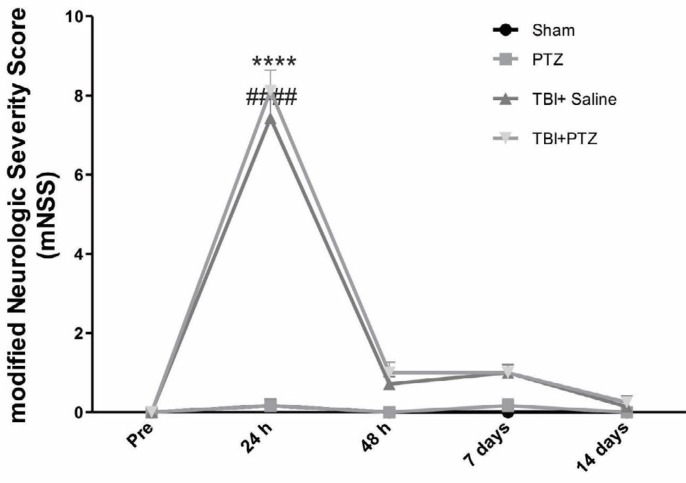
Modified neurologic severity score (mNSS) following mild traumatic brain injury (TBI). Sham group underwent anesthesia and skin incision only without TBI, and pentylenetetrazole (PTZ) group only received an intraperitoneal injection of a sub-convulsive dose of PTZ without induction of TBI. mNSS in the TBI+PTZ and TBI+Saline groups increased 24 hours after induction of mild TBI, whereas mNSS of sham and PTZ groups remained at the lowest levels compared to other TBI-induced groups. Values are expressed as mean±SEM. * indicates *P*<0.001

A significant higher mNSS was observed 24 hours after mild TBI (mean mNSS values: 8.125 for TBI+PTZ group, 7.42 for TBI+saline group versus 0 for non- traumatized groups, *P*<0.001). Impairment of neurologic functions, however, was significantly reduced 48 hr after TBI, and mNSS were returned to pre-TBI conditions 2 weeks after TBI. There were no significant differences in mNSS between TBI+PTZ and TBI+saline groups following TBI. Neurologic scores of sham and PTZ groups did not change in all observations during the 15 days of assessment (Figure 1).

Seizure behavior and epileptiform discharges increased seizure susceptibility in mice 30 days after fluid percussion injury.

**Figure 2:**
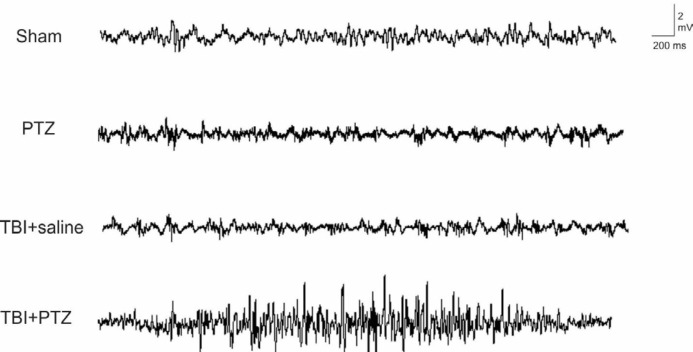
Induction of epileptiform activity by injection of pentylenetetrazole (PTZ) 15 days after traumatic brain injury (TBI) in rat brain. Typical brain electrical activities recorded through electrocorticogram (ECoG) in sham, PTZ, TBI+saline, and TBI+PTZ groups are represented. Analyses of ECoG recording showed a high number of spike and wave discharges (SWDs) in TBI+PTZ group (*P*<0.001), while the number of epileptiform activities was zero in the sham, PTZ and TBI+saline groups

Fifteen days after TBI or sham operation, a sub-convulsive dose of PTZ or saline was injected intraperitoneally in sham, PTZ, TBI+PTZ, and TBI+saline rats and seizure behavior parameters as well as electrocorticogram were evaluated. Sham-operated, PTZ, and TBI+saline rats did not display any seizure behavior or SWD through ECoG recording (Figure 2). Whereas post-traumatic cellular and molecular alterations may produce sub-clinical hyperexcitable sites throughout the brain, which are not detectable in the clinic as seizure; hence, ECoG was recorded in order to detect any sub-clinical excessive excitation. While, there was a high incidence (percent of animals experienced tonic-clonic seizure) of seizure in traumatized animals underwent PTZ challenging test (TBI+PTZ group, 83.3%; n=12, *P*<0.001). Behaviorally and electrically no seizures were observed in TBI+saline-treated animals (TBI+saline) or PTZ-treated animals (Table 1). Otherwise, neither sub-convulsive PTZ nor TBI lonely could induce seizure behavior or epileptiform discharges. Therefore, there were no differences between these groups. Most traumatized rats of TBI+PTZ group obtained the highest score and (F_ 3, 24_=12.55 , *P*<0.001) exhibited tonic-clonic seizure episodes preceded by myoclonic jerks with lower latency between PTZ injection and seizure as well as the higher mean duration of seizure attacks (F_ 3, 24_=10.59, *P*<0.001). Similarly, analyses of ECoG recording showed a high number of SWDs in TBI+PTZ group (F_ 3, 24_=15.91, *P*<0.001), while the number of epileptiform activities was zero in the sham, PTZ and TBI+saline groups (Figure 2, ). In the ECoG analyses, shortened latency, the time between injecting of PTZ and first SWD, was observed in the TBI+PTZ group (F_ 3, 24_=397, *P*<0.001) compared to the all other groups. Furthermore, amplitude (F_ 3, 24_=18.29, *P*<0.001) and duration (F_ 3, 24_=4.038, *P*<0.001) of epileptiform activities in the TBI+PTZ group were significantly different from other groups indicating lack of epileptiform activity. 

***Cellular apoptosis***

**Figure 3:**
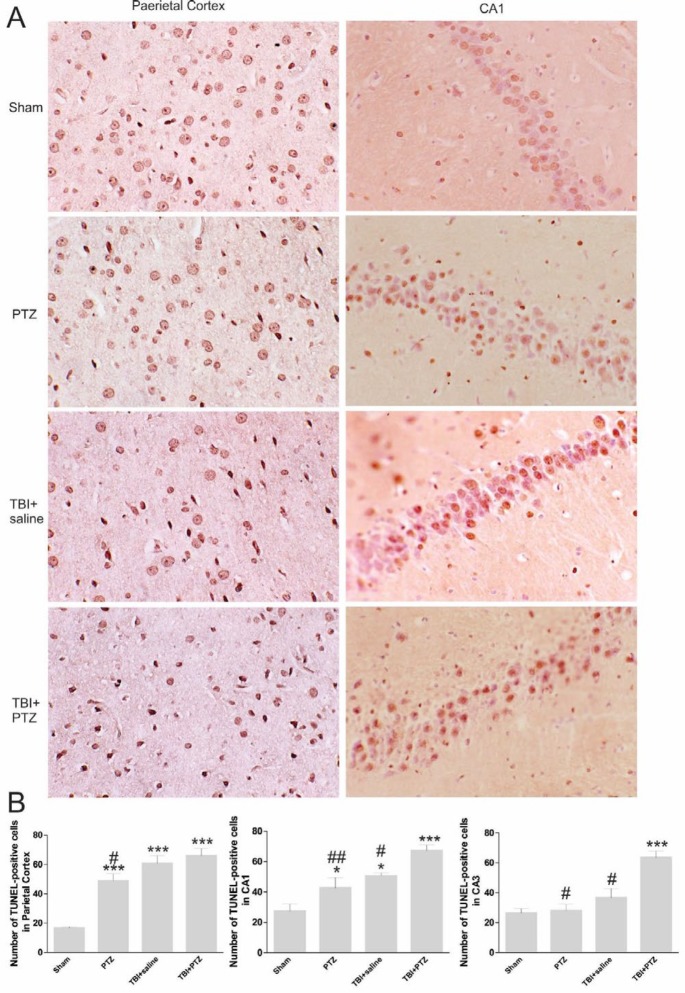
Evaluation of the effect of seizure attacks induced by intraperitoneal injection of pentylenetetrazole (PTZ) after induction of mild traumatic brain injury (TBI) on cell apoptosis in the parietal cortex and the hippocampal CA1 and CA3 areas. A: Photomicrographs of terminal deoxynucleotidyl transferase-mediated dUTP nick end labeling (TUNEL)-positive cells in the parietal cortex and the hippocampal CA1 and CA3 regions of sham, PTZ, TBI+saline, and TBI+PTZ rats 15 days after induction of TBI (40x magnification). B: Bar graphs show the mean number of TUNEL-positive cells in the parietal cortex, and the hippocampal CA1 and CA3 regions in different animal groups. The occurrence of seizure attacks induced by PTZ significantly increased the mean number of apoptotic cells in all investigated brain regions. The data are represented as mean±SEM. *** and ### indicate *P*<0.001, ##, *P*<0. 01.and * and # , *P*<0.05

The mean number of TUNEL-positive cells fifteen days after trauma in the ipsilateral parietal cortex (F_ 3, 24_=23.55, *P<*0.001) as well as the hippocampal CA1 (F_ 3, 24_=10.85, *P*<0.05) regions of TBI+saline group increased compared to the sham group (Figure 3, A and B). In addition, administration of PTZ and occurrence of seizure attacks in the TBI+PTZ group led to the appearance of an even higher number of apoptotic cells in all investigated brain regions compared to all other experimental groups (Figure 3, A and B). Intraperitoneal injection of PTZ without TBI also significantly increased the number of TUNEL-positive cells in the parietal cortex (F_ 3, 24_=23.55, *P*<0.001) and CA1 hippocampal area (F_ 3, 24_=10.85, *P*<0.05) compared to sham rats (Figure 3, A and B).

***Changes in apoptotic markers***

**Figure 4:**
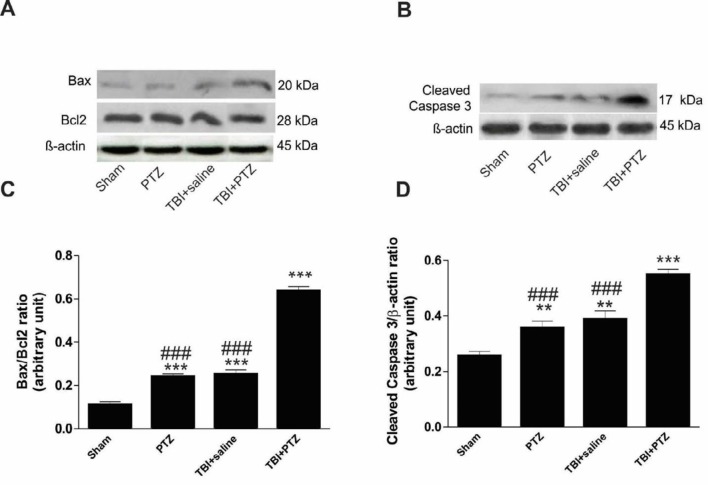
Hippocampal Bax/Bcl2 ratio and cleaved Caspase 3 levels assessed 15 days after induction of traumatic brain injury (TBI). A and B: Representative photographs of the Western blot show Bax/Bcl2 ratio and Caspase 3 in sham, pentylenetetrazole (PTZ), TBI+saline, and TBI+PTZ groups. C and D: Bar graphs indicate significant increases of Bax/Bcl2 ratio as well as the level of Caspase 3 after injection of a sub-convulsive dose of PTZ in TBI rats. Data are represented as mean±SEM. *** indicates *P*<0.001

Level of Bax, Bcl2 and Caspase 3 were investigated in the hippocampus ipsilateral to TBI site in different animal groups. Induction of TBI, followed by injection of sub-convulsive doses of PTZ, significantly increased the ratio of Bax/Bcl2 (F_ 3, 19_=458, *P*<0.001) compared to sham animals (Figure 4, A and C). In line with an increased number of apoptotic cells in TBI+PTZ rats, the ratio of Bax/Bcl2 was significantly higher in the TBI+PTZ group compared to sham, PTZ, and PTZ+saline animals (Figure 4, A and C). Furthermore, the level of Caspase 3 was significantly increased in TBI+PTZ animals (F_ 3, 19_=33.3, *P*<0.001) compared to sham, PTZ, and TBI+saline groups, suggesting an involvement of the Caspase-dependent pathway in the enhancement of apoptosis after mild TBI and seizure (Figure 4, B and D). The level of Caspase 3 was also higher in PTZ and TBI+saline groups compared to sham animals (Figure 4, B and D).

***Changes in NF-κB and Nrf2***

**Figure 5:**
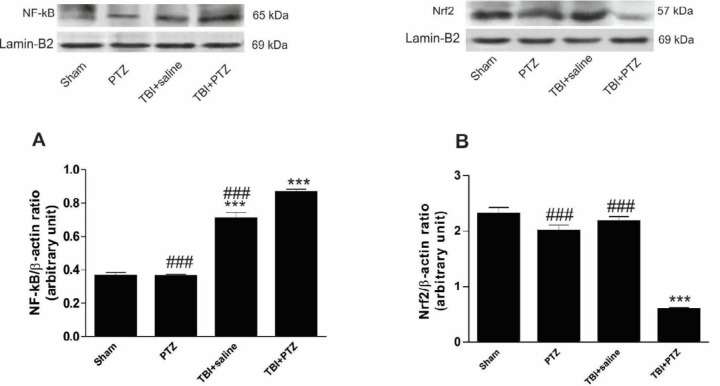
Analysis of necrosis factor kappa B (NF-κB) and nuclear factor (erythroid-derived 2)-like 2 (Nrf2) levels assessed 15 days after induction of traumatic brain injury (TBI). A and B: Representative photographs and of the Western blot show NF-κB and Nrf2 protein levels in the sham, pentylenetetrazole (PTZ), TBI+saline, and TBI+PTZ groups. C and D: Bar graphs show that NF-κB protein level was significantly higher in PTZ+TBI rats than in other animal groups. The level of NF-κB protein also increased after TBI in TBI+saline rats compared to PTZ and sham groups. In contrast to NF-κB, TBI alone did not affect the level of Nrf2 compared to PTZ and sham groups. However, intraperitoneal injection of PTZ and occurrence of seizures significantly decreased Nrf2 levels compared to all other animal groups. The data are represented as mean±SEM. *** indicates *P*<0.001

Application of sub-convulsive dose of PTZ did not change the level of NF-κB compared to sham rats (Figure 5, A and C). However, mild TBI significantly increased the level of NF-κB (F_ 3, 19_=388.1, *P*<0.001) compared to the sham and PTZ groups. Further significant enhancement of NF-κB level was observed after injection of sub-convulsive doses of PTZ after induction of mild TBI (Figure 5, A and C). 

As shown in Figure 5, the level of Nrf2 did not change in PTZ and TBI+saline groups compared to the sham group. However, Nrf2 level was significantly decreased after seizure occurrence following mild TBI (F_ 3, 19_= 61.4, *P*<0.001) (TBI+PTZ group; Figure 5, B and D).

## Discussion

Hyperexcitability of the neuronal network after moderate to severe TBI following a latent period has been shown in the cerebral cortex and hippocampus (12). Parallel to the results of previous studies, the current study also confirmed that mild TBI increases the cerebral susceptibility to late PTZ-evoked seizures after trauma. Hence, application of a sub-convulsive dose of PTZ 2 weeks after brain injury (TBI+PTZ group), led to post-traumatic tonic-clonic seizures as well as epileptiform discharges in the ECoG recordings. (12, 34, 35). The reasons for increased seizure susceptibility after TBI are multifactorial. The divergent vulnerability of neurons to brain insults results in selective death among neuronal populations during the secondary injury of TBI (13-16). In other words, certain neuronal populations are highly vulnerable. For instance, neuronal populations within the dentate gyrus, as well as other hippocampal and cortical regions are more sensitive to brain injuries (12). Therefore, this phase of damage may affect specific neuronal circuits and perturb balance between inhibitory and excitatory connections in favor of hyperexcitability of neuronal networks and may evoke spontaneous seizures (17). Using PTZ application, increased seizure susceptibility in mice has been revealed at 30 days after moderate to severe TBI (36). In addition, alterations in blood-brain barrier permeability have been shown to contribute potentially to PTE in both clinical and experimental studies (8, 37). Based on our unpublished work, the present model of TBI disrupts the blood-brain barrier integrity. Moreover, activation of inflammatory cascades in the secondary phase of TBI also contributes to the high incidence of PTE in the post-traumatic brain tissue (38).

We utilized a clinical scale to score seizure activity following the injection of a subthreshold dose of PTZ (28). Compared to all other groups where little or no evidence of seizure activity were behaviorally observed, animals 2 weeks following mild weight drop model of brain injury commonly showed evidence of seizure activity under PTZ challenging test that could be qualitatively assessed in terms of the animals’ behavior (TBI+PTZ group). Correlated to these clinical measures of seizure activity, post-traumatic brains showed abnormal hyperexcitability following a single dose of PTZ in electrophysiological measurements. 

Our results demonstrate that the traumatized brain is endangered to adverse outcomes of this PTE. As a consequence, we found that these induced seizures significantly intensified cellular damage observed in the histopathological and molecular assessments. Whereas aggravation of histopathological damage following an induced period of PTE is likely due to multiple mechanisms (12), compromise of the blood-brain barrier can itself induce epileptiform discharges, as well as seizures (39-42). Indeed, periods of PTE have been reported to reduce blood flow in local brain regions that are already at risk of ischemia due to brain trauma (37). While, upraised seizure susceptibility in the course of secondary damage leads to seizure attacks following application of a sub-convulsive dose of PTZ, seizures, in turn, deteriorate neuronal loss in different brain regions by activating the mitochondrial and Caspase- related apoptotic pathways (43). In keeping with our findings, application of sub-convulsive doses of PTZ has also been shown to induce apoptosis and to activate Caspase 3 possibly due to GABAergic disinhibition (44). The additive or synergistic effects of seizure and mild TBI potentiate their morphological or molecular effects on the brain and markedly increase the number of DNA fragmentation in the hippocampus and neocortex. Adverse degenerative consequences of seizure activity after TBI have been shown to deteriorate the neurological outcome by exacerbating the primary damage of TBI (2, 45). The induction of convulsion by low concentrations of kainic acid after TBI caused a loss of ipsilateral hippocampal CA3, CA4, and hilar neurons following increased metabolic rates and neuronal activity in the hippocampus (19). The Caspase and Bcl2 family proteins play a crucial role in neuronal death after seizures and epileptogenesis in several models of brain injury (46). 

NF-κB and Nrf2 play important roles in the neuroinflammation and cell survival cascades following TBI (47, 48). NF-κB is a transcriptional factor required for the gene expression of several inflammatory mediators and plays a crucial role in the development of pathological processes in both epilepsy and TBI (38, 49). Levels of NF-κB activity are increased in neuroglial cells within hours of TBI and remains high for many months, suggesting a role for NF-κB in the epilepsy-related inflammatory process (50). Our results indicated an increased level of NF-κB after mild TBI (TBI+saline group); an effect that was significantly amplified by seizures (TBI+PTZ group). NF-κB regulates seizure threshold and gene transcription following convulusion (51). 

Nrf2, a neuroprotective transcription factor, promotes the expression of numerous antioxidant and antiinflammatory proteins and may play protective roles in epilepsy (52) and TBI (53). Activation of the Nrf2 pathway by small molecules or by overexpression has been shown to promote the expression of numerous antioxidants (52) and to slow down neuronal loss in a range of pathological conditions, including TBI (54). As, Nrf2 plays a protective role in acquired epilepsy (55, 56), Nrf2, as well as its downstream gene products have been suggested as promising anti-epileptic or anti-epileptogenic therapies (56). Our novel finding revealed that mild TBI did not change the level of Nrf2. However, Nrf2 values were significantly decreased after convulsions in traumatized rats (TBI+PTZ group). This suggests that endogenous antioxidant defense mechanisms of Nrf2 are important in preventing the brain from the exitotoxic condition. 

**Table 1:** Seizure behavior parameters and ECoG findings following an induced PTS in the rat model

Seizure behavior					Eclectrocorticogram				
	Sham	PTZ	TBI+Saline	TBI+PTZ		Sham	PTZ	TBI+Saline	TBI+PTZ
Incidence (%)	0	0	0	83	Number	0	0	0	22.8±12^***^
Score	0	0.6 ±1.2	0±0.0	4.2±1.4^***^	Latency (min)	60±0.0	60±0.0	60±0.0	9.01±6.05^***^
Latency (min)	60±0.0	60±0.0	60±0.0	12.73±3.2 ^***^	Duration (mS)	0	0	0	0.012±0.1^***^
Duration (min)	0±0.0	4.16±10	0±0.0	25.818± 2.28 ^***^	Amplitude (mV)	0	0	0±0.0	0.75±0.38 ^***^

PTZ: Pentylenetetrazole; ECoG: Electrocorticogram; PTS: Post-traumatic seizures; mS: milli second; mS: milli volt. ^***^ *P*<0.001 compared with sham

## Conclusion

Overall, our data highlight the extreme vulnerability of the post-traumatic brain to the patterns of abnormal neuronal hyperexcitability. Accordingly, seizure occurrence following mild TBI aggravates cell injury via activation of neuroinflammatory or apoptotic processes and may increase the risk of PTE. Since, numerous catastrophic mechanisms such as excessive excitation gradually emerge after head trauma, novel drugs or intervention strategies during the latent period of TBI must be applied to control the secondary devastating cascades. Given the extremely diverse set of target genes thought to be involved in secondary insult, targeting these molecules may constitute a promising novel therapeutic approach to prevent adverse outcomes like PTE in the future. 

## References

[1] Frye CA, Scalise TJ (2000). Anti-seizure effects of progesterone and 3alpha,5alpha-THP in kainic acid and perforant pathway models of epilepsy. Psychoneuroendocrinology.

[2] Asikainen I, Kaste M, Sarna S (1999). Early and late posttraumatic seizures in traumatic brain injury rehabilitation patients: brain injury factors causing late seizures and influence of seizures on long-term outcome. Epilepsia.

[3] Angeleri F, Majkowski J, Cacchio G, Sobieszek A, D’Acunto S, Gesuita R (1999). Posttraumatic epilepsy risk factors: one-year prospective study after head injury. Epilepsia.

[4] Englander J, Bushnik T, Duong TT, Cifu DX, Zafonte R, Wright J (2003). Analyzing risk factors for late posttraumatic seizures: a prospective, multicenter investigation. Arch Phys Med Rehabil.

[5] Eftekhar B, Sahraian MA, Nouralishahi B, Khaji A, Vahabi Z, Ghodsi M (2009). Prognostic factors in the persistence of posttraumatic epilepsy after penetrating head injuries sustained in war. J Neurosurg.

[6] Salazar AM, Jabbari B, Vance SC, Grafman J, Amin D, Dillon JD (1985). Epilepsy after penetrating head injury I Clinical correlates: a report of the Vietnam Head Injury Study. Neurology.

[7] Bao YH, Bramlett HM, Atkins CM, Truettner JS, Lotocki G, Alonso OF (2010). Post-traumatic seizures exacerbate histopathological damage after fluid-percussion brain injury. J Neurotrauma.

[8] D’Ambrosio R, Perucca E (2004). Epilepsy after head injury. Curr Opin Neurol.

[9] Pitkanen A, Bolkvadze T (2012). Head Trauma and Epilepsy. Jasper’s Basic Mechanisms of the Epilepsies [Internet].

[10] Khodaie B, Lotfinia AA, Ahmadi M, Lotfinia M, Jafarian M, Karimzadeh F (2015). Structural and functional effects of social isolation on the hippocampus of rats with traumatic brain injury. Behav Brain Res.

[11] Jafarian M, Karimzadeh F, Alipour F, Attari F, Lotfinia AA, Speckmann EJ (298). Cognitive impairments and neuronal injury in different brain regions of a genetic rat model of absence epilepsy. Neuroscience.

[12] Vespa PM, McArthur DL, Xu Y, Eliseo M, Etchepare M, Dinov I (2010). Nonconvulsive seizures after traumatic brain injury are associated with hippocampal atrophy. Neurology.

[13] Gao X, Deng-Bryant Y, Cho W, Carrico KM, Hall ED, Chen J (2008). Selective death of newborn neurons in hippocampal dentate gyrus following moderate experimental traumatic brain injury. J Neurosci Res.

[14] Grady MS, Charleston JS, Maris D, Witgen BM, Lifshitz J (2003). Neuronal and glial cell number in the hippocampus after experimental traumatic brain injury: analysis by stereological estimation. J Neurotrauma.

[15] Conti AC, Raghupathi R, Trojanowski JQ, McIntosh TK (1998). Experimental brain injury induces regionally distinct apoptosis during the acute and delayed post-traumatic period. J Neurosci.

[16] Bigford GE, Alonso OF, Dietrich D, Keane RW (2009). A novel protein complex in membrane rafts linking the NR2B glutamate receptor and autophagy is disrupted following traumatic brain injury. J Neurotrauma.

[17] Herman ST (2006). Clinical trials for prevention of epileptogenesis. Epilepsy Res.

[18] Golarai G, Greenwood AC, Feeney DM, Connor JA (2001). Physiological and structural evidence for hippocampal involvement in persistent seizure susceptibility after traumatic brain injury. J Neurosci.

[19] Zanier ER, Lee SM, Vespa PM, Giza CC, Hovda DA (2003). Increased hippocampal CA3 vulnerability to low-level kainic acid following lateral fluid percussion injury. J Neurotrauma.

[20] Sherer M, Struchen MA, Yablon SA, Wang Y, Nick TG (2008). Comparison of indices of traumatic brain injury severity: Glasgow Coma Scale, length of coma and post-traumatic amnesia. J Neurol Neurosurg Psychiatry.

[21] Coulter DA, Rafiq A, Shumate M, Gong QZ, DeLorenzo RJ, Lyeth BG (1996). Brain injury-induced enhanced limbic epileptogenesis: anatomical and physiological parallels to an animal model of temporal lobe epilepsy. Epilepsy Res.

[22] Nilsson P, Ronne-Engstrom E, Flink R, Ungerstedt U, Carlson H, Hillered L (1994). Epileptic seizure activity in the acute phase following cortical impact trauma in rat. Brain Res.

[23] Reeves TM, Lyeth BG, Phillips LL, Hamm RJ, Povlishock JT (1997). The effects of traumatic brain injury on inhibition in the hippocampus and dentate gyrus. Brain Res.

[24] Statler KD, Swank S, Abildskov T, Bigler ED, White HS (2008). Traumatic brain injury during development reduces minimal clonic seizure thresholds at maturity. Epilepsy Res.

[25] Ghadiri T, Sharifzadeh M, Khodagholi F, Modarres Mousavi SM, Hassanzadeh G, Zarrindast MR (2014). A novel traumatic brain injury model for induction of mild brain injury in rats. J Neurosci Methods.

[26] Yuan XQ, Prough DS, Smith TL, Dewitt DS (1988). The effects of traumatic brain injury on regional cerebral blood flow in rats. J Neurotrauma.

[27] Xiong Y, Mahmood A, Meng Y, Zhang Y, Qu C, Schallert T (113). Delayed administration of erythropoietin reducing hippocampal cell loss, enhancing angiogenesis and neurogenesis, and improving functional outcome following traumatic brain injury in rats: comparison of treatment with single and triple dose. J Neurosurg.

[28] Bao YH, Bramlett HM, Atkins CM, Truettner JS, Lotocki G, Alonso OF (2011). Post-traumatic seizures exacerbate histopathological damage after fluid-percussion brain injury. J Neurotrauma.

[29] Iannone M, Cosco D, Cilurzo F, Celia C, Paolino D, Mollace V (2010). A novel animal model to evaluate the ability of a drug delivery system to promote the passage through the BBB. Neurosci Lett.

[30] Kong S, Qian B, Liu J, Fan M, Chen G, Wang Y (2010). Cyclothiazide induces seizure behavior in freely moving rats. Brain Res.

[31] Otsuki Y, Li Z, Shibata MA (2003). Apoptotic detection methods--from morphology to gene. Prog Histochem Cytochem.

[32] Bradford MM (1976). A rapid and sensitive method for the quantitation of microgram quantities of protein utilizing the principle of protein-dye binding. Anal Biochem.

[33] Vakilzadeh G, Khodagholi F, Ghadiri T, Darvishi M, Ghaemi A, Noorbakhsh F (2014). Protective effect of a cAMP analogue on behavioral deficits and neuropathological changes in Cuprizone model of demyelination. Mol Neurobiol.

[34] Atkins CM, Truettner JS, Lotocki G, Sanchez-Molano J, Kang Y, Alonso OF (2010). Post-traumatic seizure susceptibility is attenuated by hypothermia therapy. Eur J Neurosci.

[35] Kharatishvili I, Pitkanen A (2010). Association of the severity of cortical damage with the occurrence of spontaneous seizures and hyperexcitability in an animal model of posttraumatic epilepsy. Epilepsy Res.

[36] Mukherjee S, Zeitouni S, Cavarsan CF, Shapiro LA (2013). Increased seizure susceptibility in mice 30 days after fluid percussion injury. Front Neurol.

[37] D’Ambrosio R, Fairbanks JP, Fender JS, Born DE, Doyle DL, Miller JW (2004). Post-traumatic epilepsy following fluid percussion injury in the rat. Brain.

[38] Jayakumar AR, Tong XY, Ruiz-Cordero R, Bregy A, Bethea JR, Bramlett HM (2014). Activation of NF-kappaB mediates astrocyte swelling and brain edema in traumatic brain injury. J Neurotrauma.

[39] van Vliet EA, Aronica E, Gorter JA (2014). Role of blood-brain barrier in temporal lobe epilepsy and pharmacoresistance. Neuroscience.

[40] van Vliet EA, da Costa Araujo S, Redeker S, van Schaik R, Aronica E, Gorter JA (2007). Blood-brain barrier leakage may lead to progression of temporal lobe epilepsy. Brain.

[41] van Vliet EA, Otte WM, Wadman WJ, Aronica E, Kooij G, de Vries HE (2016). Blood-brain barrier leakage after status epilepticus in rapamycin-treated rats I: Magnetic resonance imaging. Epilepsia.

[42] van Vliet EA, van Schaik R, Edelbroek PM, Voskuyl RA, Redeker S, Aronica E (2007). Region-specific overexpression of P-glycoprotein at the blood-brain barrier affects brain uptake of phenytoin in epileptic rats. J Pharmacol Exp Ther.

[43] Sedlak TW, Oltvai ZN, Yang E, Wang K, Boise LH, Thompson CB (1995). Multiple Bcl-2 family members demonstrate selective dimerizations with Bax. Proc Natl Acad Sci U S A.

[44] Naseer MI, Shupeng L, Kim MO (2009). Maternal epileptic seizure induced by pentylenetetrazol: apoptotic neurodegeneration and decreased GABAB1 receptor expression in prenatal rat brain. Mol Brain.

[45] Mazzini L, Cossa FM, Angelino E, Campini R, Pastore I, Monaco F (2003). Posttraumatic epilepsy: neuroradiologic and neuropsychological assessment of long-term outcome. Epilepsia.

[46] Henshall DC, Simon RP (2005). Epilepsy and apoptosis pathways. J Cereb Blood Flow Metab.

[47] Yan W, Wang HD, Hu ZG, Wang QF, Yin HX (2008). Activation of Nrf2-ARE pathway in brain after traumatic brain injury. Neurosci Lett.

[48] Digicaylioglu M, Lipton SA (2001). Erythropoietin-mediated neuroprotection involves cross-talk between Jak2 and NF-kappaB signalling cascades. Nature.

[49] Kaltschmidt B, Kaltschmidt C (2009). NF-kappaB in the nervous system. Cold Spring Harb Perspect Biol.

[50] Mattson MP, Camandola S (2001). NF-kappaB in neuronal plasticity and neurodegenerative disorders. J Clin Invest.

[51] Lubin FD, Ren Y, Xu X, Anderson AE (2007). Nuclear factor-kappa B regulates seizure threshold and gene transcription following convulsant stimulation. J Neurochem.

[52] Mazzuferi M, Kumar G, van Eyll J, Danis B, Foerch P, Kaminski RM Nrf2 defense pathway: Experimental evidence for its protective role in epilepsy. Ann Neurol.

[53] Miller DM, Singh IN, Wang JA, Hall ED (2014). Nrf2-ARE activator carnosic acid decreases mitochondrial dysfunction, oxidative damage and neuronal cytoskeletal degradation following traumatic brain injury in mice. Exp Neurol.

[54] Xiong W, MacColl Garfinkel AE, Li Y, Benowitz LI, Cepko CL (2015). NRF2 promotes neuronal survival in neurodegeneration and acute nerve damage. J Clin Invest.

[55] Mazzuferi M, Kumar G, van Eyll J, Danis B, Foerch P, Kaminski RM (2013). Nrf2 defense pathway: Experimental evidence for its protective role in epilepsy. Ann Neurol.

[56] Patel M (2015). Nrf2 to the rescue. Epilepsy Curr.

